# Supplementary material for: Genetic risk for autoimmunity is associated with distinct changes in the human gut microbiome
Source: Nat Commun. 2019 Aug 9;10:3621. doi: 10.1038/s41467-019-11460-x (PMC6689114; doi:10.1038/s41467-019-11460-x)
Supplement: Supplementary file 1 — Supplementary Info [file 41467_2019_11460_MOESM1_ESM.pdf]

Supplemental Information

Genetic risk for autoimmunity is associated with distinct changes in the human gut microbiome

Russell et al.

Genotype	Number of subjects	T1D Mother	T1D Father	T1D Siblings	T1D Grandparent	Male;Female	Antibiotics (>11 months)	Breastfed (>11 months)
High	30							
DR3/4	30	-	-	-	3	18;12	2	1
Increased	99							
DR3/Z	43	-	-	-	2	24;19	2	2
DR4/Z	56	-	1	-	6	27;29	4	6
Neutral	134	-	-	-	-			
DR3/X	45	-	1	1	1	22;23	3	7
DR4/X	47	-	-	-	2	27;20	5	6
DRZ/Z	42	-	1	-	4	20;22	3	4
Decreased	140							
DRX/X	140	3	3	1	6	73;67	11	17
Total	403	3	6	2	24	403	30	43

X = protective haplotype

Z = non-risk and non-protective haplotype

Supplementary Table 1 – Dataset characteristics

Dataset characteristics including number of participants per genetic risk group category, their genotype, sex, number of participants with T1D afflicted family members included in the analysis, number of participants who took antibiotics 1 month prior to sample collection and the number of participants still breastfed within 1 month of sample collection.
